# Serum monocyte fraction of white blood cells is increased in patients with high Gleason score prostate cancer

**DOI:** 10.18632/oncotarget.13052

**Published:** 2016-11-03

**Authors:** Takuji Hayashi, Kazutoshi Fujita, Go Tanigawa, Atsunari Kawashima, Akira Nagahara, Takeshi Ujike, Motohide Uemura, Tetsuya Takao, Seiji Yamaguchi, Norio Nonomura

**Affiliations:** ^1^ Department of Urology, Osaka University Graduate School of Medicine, Suita, Japan; ^2^ Department of Urology, Osaka General Medical Center, Osaka, Japan

**Keywords:** serum, monocyte, Gleason score, prostate cancer, biopsy

## Abstract

Systemic inflammation and immune responses are reported to be associated with progressive prostate cancer. In this study, we explored which among the fractions of white blood cell (WBC) and C-reactive protein (CRP) level were associated with high Gleason score prostate cancer. Prostate needle biopsy was performed in 966 men with suspicion of prostate cancer. We assessed age, serum prostate-specific antigen (PSA), prostate volume, WBC count, fractions of WBCs (neutrophils, lymphocytes, monocytes, basophils, and eosinophils), and CRP level before biopsy for associations with biopsy findings. Among all men, 553 (57.2%) were positive for prostate cancer including 421 with high Gleason score cancer (Gleason score ≥7). Age, PSA, PSA density (PSAD), serum monocyte fraction of WBC, monocyte-to-lymphocyte ratio (MLR), and CRP were significantly associated with high Gleason score cancer (*p*<0.01). Multivariate analysis showed that age, PSA, PSAD, and serum monocyte fraction were significantly associated with high Gleason score prostate cancer (*p* <0.01). In 571 patients with PSA of <10 ng/ml, age, PSA, PSAD, serum WBC count, neutrophil fraction, monocyte fraction, and MLR were significantly associated with high Gleason score prostate cancer (*p*<0.05). Multivariate analysis showed that age, PSAD, and serum monocyte fraction were significantly associated with high Gleason score prostate cancer (*p*<0.01). The monocyte fraction of WBCs was increased in patients with high Gleason score prostate cancer, suggesting an interaction of monocytes with the progression of prostate cancer.

## INTRODUCTION

Serum prostate-specific antigen (PSA) level has been widely used to detect prostate cancer and monitor its treatment, including surgical therapy, radiotherapy, hormonal therapy, chemotherapy, and active surveillance. However, it is difficult to predict the aggressiveness of prostate cancer using the PSA measurement alone. [1]

Although several serum or urine biomarkers for higher sensitivity and specificity of detection of prostate cancer have emerged, [2, 3] the problem of overdiagnosis and overtreatment of clinically insignificant prostate cancer remains unresolved. To formulate an appropriate differential diagnosis between clinically significant and insignificant prostate cancer, we need convenient biomarkers for the prediction of pathological high Gleason score cancer (Gleason score of ≥7) before needle biopsy. We reported that urinary fucosylated PSA is one of the new biomarkers for the detection of high Gleason score cancer. [4]

It is important to elucidate the mechanism of systemic inflammation and immune responses to prostate cancer. We also reported that low serum neutrophil count predicts a positive prostate biopsy [5] and that the white blood cell (WBC) count is associated with benign prostatic hyperplasia. [6] Two meta-analyses reported that C-reactive protein (CRP) may also have prognostic value in patients with prostate cancer. [7, 8] Because the WBC count and the fractions of each of the WBC types and the CRP level are indicators of systemic inflammation and immune responses, these counts, ratios, or levels may be good candidate biomarkers for predicting aggressive cancer. It was reported that the serum neutrophil-to-lymphocyte ratio (NLR) predicts prostate cancer in men undergoing needle biopsy [9] and that the serum monocyte-to-lymphocyte ratio (MLR) predicts poor prognosis in primary pulmonary lymphoepithelioma-like carcinoma. [10]

The aim of this study was therefore to explore which parameters among serum WBC count and the fractions of WBCs and CRP and NLR and MLR are associated with high Gleason score prostate cancer.

## RESULTS

The characteristics and biopsy findings of all 966 men are summarized in Table 1. Of them, 553 (57.2%) were positive for prostate cancer including 422 with high Gleason score cancer.

**Table 1:** Characteristics and biopsy findings of all men (comparison among three groups)

Characteristic	NC		LG		HG		*p*-Value
Number	413		131		422		
**Age (years)**	**67**	**(44-84)**	**69**	**(36-87)**	**72**	**(43-89)**	**<0.0001**
**PSA (ng/ml)**	**6.39**	**(0.36-935)**	**7.53**	**(1.78-126.8)**	**13.29**	**(1.49-10138)**	**<0.0001**
**Prostate volume (cm**^3^)	**40.0**	**(10.0-150.0)**	**28.8**	**(7.3-103.0)**	**28.0**	**(7.8-160.0)**	**<0.0001**
**PSAD (ng/ml/cm**^3^)	**0.17**	**(0.01-12.99)**	**0.24**	**(0.03-2.92)**	**0.50**	**(0.04-330.87)**	**<0.0001**
WBC count (/μl)	5850	(2390-13870)	5670	(3530-12400)	5705	(3170-12000)	0.3237
Neutrophil fraction (%)	61.2	(34.2-91.5)	60.7	(27.0-87.3)	60.0	(36.0-80.9)	0.2985
Lymphocyte fraction (%)	28.8	(4.2-55.8)	27.2	(10.4-65.4)	28.2	(9.2-55.1)	0.7471
**Monocyte fraction (%)**	**6.7**	**(2.5-13.2)**	**6.7**	**(1.8-17.1)**	**7.6**	**(2.0-15.4)**	**<0.0001**
Basophil fraction (%)	0.6	(0-3.1)	0.5	(0-2.2)	0.6	(0-4.1)	0.7748
Eosinophil fraction (%)	2.5	(0-18.1)	2.2	(0-13.1)	2.4	(0-15.5)	0.4096
CRP (mg/dl)	0.05	(0-8.55)	0.05	(0-8.53)	0.05	(0-16.75)	0.2989
NLR	2.12	(0.61-21.79)	2.21	(0.41-8.39)	2.12	(0.66-8.31)	0.6996
**MLR**	**0.23**	**(0.08-1.49)**	**0.25**	**(0.08-0.87)**	**0.27**	**(0.04-1.25)**	**<0.0001**

Abbreviations: NC, no cancer; LG, low Gleason score cancer; HG: high Gleason score cancer; PSA, prostate-specific antigen; PSAD, PSA density; WBC, white blood cell; CRP, C-reactive protein; NLR, neutrophil-to-lymphocyte ratio; MLR, monocyte-to-lymphocyte ratio.

We compared the characteristics of the men with negative biopsy to those with positive biopsy (both low Gleason score and high Gleason score cancer) (Supplementary Table 1). Serum monocyte fraction was significantly higher (*p* < 0.01) in the men with positive biopsy than in those with negative biopsy. Serum monocyte fraction was one of predictors of positive biopsy in multiple logistic regression analysis (Supplementary Table 2).

In turn, we compared the characteristics of the men without clinically significant cancer (both the men with negative biopsy and those in whom low Gleason score cancer was found) to those in whom high Gleason score cancer was found (Table 2). Age, PSA, PSAD, monocyte fraction, and MLR were significantly higher (*p* < 0.01), CRP was not significantly higher (*p* = 0.146), and prostate volume was significantly lower (*p* < 0.01) in the men in whom high Gleason score prostate cancer was found than in those with negative biopsy and in those in whom low Gleason score prostate cancer was found.

**Table 2:** Characteristics and biopsy findings of all men (comparison of the men with negative biopsy and those of low Gleason score cancer to those with high Gleason score cancer)

Characteristic	NC+LG		HG		*p*-Value
Number	413+131		422		
**Age (years)**	**67**	**(36-87)**	**72**	**(43-89)**	**<0.0001**
**PSA (ng/ml)**	**6.66**	**(0.36-935)**	**13.29**	**(1.49-10138)**	**<0.0001**
**Prostate volume (cm**^3^)	**37.4**	**(7.3-150.0)**	**28.0**	**(7.8-160.0)**	**<0.0001**
**PSAD (ng/ml/cm**^3^)	**0.18**	**(0.01-12.99)**	**0.50**	**(0.04-330.87)**	**<0.0001**
WBC count (/μl)	5815	(2390-13870)	5705	(3170-12000)	0.1552
Neutrophil fraction (%)	61.1	(27.0-91.5)	60.0	(36.0-80.9)	0.1145
Lymphocyte fraction (%)	28.3	(4.2-65.4)	28.2	(9.2-55.1)	0.9602
**Monocyte fraction (%)**	**6.7**	**(1.8-17.1)**	**7.6**	**(2.0-15.4)**	**<0.0001**
Basophil fraction (%)	0.6	(0-3.1)	0.6	(0-4.1)	0.7333
Eosinophil fraction (%)	2.5	(0-18.1)	2.4	(0-15.5)	0.7467
CRP (mg/dl)	0.05	(0-8.55)	0.05	(0-16.75)	0.1460
NLR	2.13	(0.41-21.79)	2.12	(0.66-8.31)	0.5491
**MLR**	**0.24**	**(0.08-1.49)**	**0.27**	**(0.04-1.25)**	**<0.0001**

Abbreviations: NC, no cancer; LG, low Gleason score cancer; HG: high Gleason score cancer; PSA, prostate-specific antigen; PSAD, PSA density; WBC, white blood cell; CRP, C-reactive protein; NLR, neutrophil-to-lymphocyte ratio; MLR, monocyte-to-lymphocyte ratio.

Logistic regression analysis revealed that age, PSA, PSAD, serum monocyte fraction, MLR, and CRP level were predictors of high Gleason score prostate cancer, whereas the WBC count, other fractions of WBCs (neutrophils, lymphocytes, basophils, and eosinophils), and neutrophil-to-lymphocyte ratio (NLR) were not (Table 3). Multiple logistic regression analysis showed that age, PSA, PSAD, and monocyte fraction were significant predictors of high Gleason score prostate cancer.

**Table 3:** Univariate and multivariate analyses for predicting high Gleason score prostate cancer in all men (*n*=966)

	Univariate analysis			Multivariate analysis		
Variable	OR	95% CI	*p*-Value	OR	95% CI	*p*-Value
**Age (years)**	**1.08**	**(1.06-1.10)**	**<0.0001**	**1.06**	**(1.04-1.09)**	**<0.0001**
**PSA (ng/ml)**	**1.03**	**(1.02-1.04)**	**<0.0001**	**0.96**	**(0.95-0.97)**	**<0.0001**
**PSAD (ng/ml/cm**^3^)	**14.64**	**(8.30-27.14)**	**<0.0001**	**72.29**	**(30.74-181.90)**	**<0.0001**
WBC count (/μl)	1.00	(1.00-1.00)	0.160			
Neutrophil (%)	0.99	(0.97-1.00)	0.112			
Lymphocyte (%)	1.00	(0.98-1.01)	0.921			
**Monocyte (%)**	**1.27**	**(1.19-1.36)**	**<0.0001**	**1.18**	**(1.09-1.28)**	**<0.0001**
Basophil (%)	0.97	(0.68-1.38)	0.867			
Eosinophil (%)	1.01	(0.95-1.07)	0.784			
**CRP (mg/dl)**	**1.20**	**(1.06-1.39)**	**0.0019**	**―**	**―**	**―**
NLR	0.96	(0.87-1.05)	0.418			
**MLR**	**6.67**	**(2.54-18.28)**	**<0.0001**	**―**	**―**	**―**

Abbreviations: OR, odds ratio; CI, confidence interval; PSA, prostate-specific antigen; PSAD, PSA density; WBC, white blood cell; CRP, C-reactive protein; NLR, neutrophil-to-lymphocyte ratio; MLR, monocyte-to-lymphocyte ratio.

**Figure 1:**
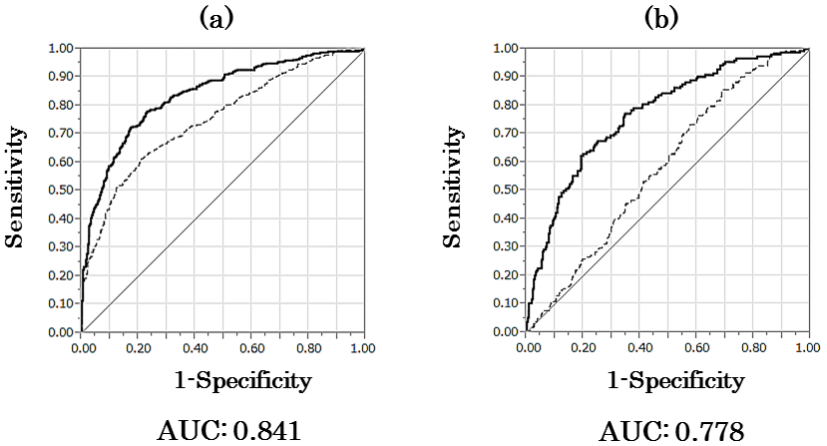
Receiver operator characteristic curves for no cancer and low Gleason score or high Gleason score cancer **a**. AUC for age, PSA, PSAD, and monocyte fraction (bold line) compared to AUC for PSA only (dotted line) to predict high Gleason score prostate cancer in all men. **b**. AUC for age, PSAD, and monocyte fraction (bold line) compared to AUC for PSA only (dotted line) to predict high Gleason score prostate cancer in men with PSA < 10 ng/ml. AUC, area under the curve; PSA, prostate-specific antigen; PSAD, PSA density.

Regression coefficients for an optimum model were estimated, and the following model predicting the probability of detecting high Gleason score cancer by biopsy was calculated: *P* = [1+exp (6.656-0.059×age+0.041×PSA-4.28×PSAD-0.166×monocyte fraction)]^-1^. We plotted the area under the receiver operator characteristics curve (AUC) using no cancer and low Gleason score cancer or high Gleason score cancer (Figure 1a). The AUC for the probability of predicting high Gleason score prostate cancer by age, PSA, PSAD, and serum monocyte fraction was 0.841 (95% confidence interval [CI] 0.814-0.865), whereas the AUC by PSA was 0.754 (95% CI 0.722-0.784) in all men. The sensitivity and specificity of the model at the best cutoff value were 77.6% and 77.9%, respectively.

The data of clinical stages are available in 349 cases of 553 positive biopsy cases. In this cohort, serum monocyte fraction of WBC was significantly higher in the patients with clinical stage ≥T3 than with ≤T2 (*p* < 0.01).

The prediction of high Gleason score prostate cancer may be more valuable for the identification of clinically significant cancer in men with a PSA < 10 ng/ml rather than in men with a higher PSA level. We performed a subanalysis of 571 men with PSA < 10 ng/ml, and the characteristics and biopsy findings of these men are shown in Table 4. Of these men, 249 (43.6%) were positive for prostate cancer including 155 with high Gleason score cancer.

**Table 4:** Characteristics and biopsy findings of men with PSA <10 ng/ml (comparison of the men with negative biopsy and those with low Gleason score cancer to those with high Gleason score cancer)

Characteristic	NC+LG		HG		*p*-Value
Number	322+94		155		
**Age (years)**	**67**	**(36-82)**	**70**	**(53-87)**	**0.0003**
**PSA (ng/ml)**	**5.76**	**(0.36-9.99)**	**6.32**	**(1.49-9.91)**	**0.0017**
**Prostate volume (cm**^3^)	**34.0**	**(7.3-150.0)**	**25.7**	**(7.8-77.0)**	**<0.0001**
**PSAD (ng/ml/cm**^3^)	**0.16**	**(0.01-0.82)**	**0.24**	**(0.04-0.81)**	**<0.0001**
**WBC count (/μl)**	**5700**	**(2390-13870)**	**5360**	**(3170-11660)**	**0.0075**
**Neutrophil fraction (%)**	**61.2**	**(27.0-91.5)**	**58.7**	**(38.0-78.0)**	**0.0090**
Lymphocyte fraction (%)	28.1	(4.2-65.4)	30.0	(9.2-48.7)	0.1475
**Monocyte fraction (%)**	**6.7**	**(1.8-13.2)**	**7.3**	**(4.9-15.4)**	**<0.0001**
**Basophil fraction (%)**	**0.6**	**(0-3.1)**	**0.7**	**(0-4.1)**	**0.0291**
Eosinophil fraction (%)	2.5	(0-15.7)	2.7	(0-14.2)	0.0653
CRP (mg/dl)	0.05	(0-6.51)	0.04	(0-6.41)	0.4967
NLR	2.16	(0.41-21.79)	1.97	(0.85-8.23)	0.0551
**MLR**	**0.23**	**(0.08-0.97)**	**0.25**	**(0.13-1.25)**	**0.0220**

Abbreviations: NC, no cancer; LG, low Gleason score cancer; HG: high Gleason score cancer; PSA, prostate-specific antigen; PSAD, PSA density; WBC, white blood cell; CRP, C-reactive protein; NLR, neutrophil-to-lymphocyte ratio; MLR, monocyte-to-lymphocyte ratio.

Age, PSA, PSAD, monocyte fraction, basophil fraction, and MLR were significantly higher (*p* < 0.05), whereas prostate volume, serum WBC count, and serum neutrophil fraction were significantly lower (*p* < 0.01) in the men in whom high Gleason score prostate cancer was found than in those with negative biopsy and in those in whom low Gleason score prostate cancer was found. CRP was not associated with the difference among these groups.

Logistic regression analysis revealed that age, PSA, PSAD, serum WBC count, neutrophil fraction, monocyte fraction, and MLR were significantly associated with high Gleason score prostate cancer, whereas other fractions of WBCs (lymphocytes, basophils, and eosinophils), CRP level, and NLR were not (Table 5). Multiple logistic regression analysis showed that age, PSAD, and serum monocyte fraction were significant predictors of high Gleason score prostate cancer.

**Table 5:** Univariate and multivariate analyses for predicting high Gleason score prostate cancer in men with PSA <10 ng/ml (*n* = 571)

	Univariate analysis			Multivariate analysis		
Variable	OR	95% CI	*p*-Value	OR	95% CI	*p*-Value
**Age (years)**	**1.06**	**(1.03-1.09)**	**0.0001**	**1.05**	**(1.02-1.08)**	**0.0014**
**PSA (ng/ml)**	**1.17**	**(1.06-1.29)**	**0.0010**	**―**	**―**	**―**
**PSAD (ng/ml/cm**^3^)	**884.44**	**(160.65-5468.38)**	**<0.0001**	**857.33**	**(149.36-5595.16)**	**<0.0001**
**WBC count (/μl)**	**1.00**	**(1.00-1.00)**	**0.0177**	**―**	**―**	**―**
**Neutrophil (%)**	**0.97**	**(0.95-1.00)**	**0.0113**	**―**	**―**	**―**
Lymphocyte (%)	1.01	(0.99-1.03)	0.339			
**Monocyte (%)**	**1.32**	**(1.19-1.46)**	**<0.0001**	**1.30**	**(1.17-1.45)**	**<0.0001**
Basophil (%)	1.59	(1.00-2.57)	0.0544			
Eosinophil (%)	1.06	(0.98-1.15)	0.124			
CRP (mg/dl)	1.16	(0.88-1.53)	0.279			
NLR	0.89	(0.75-1.04)	0.148			
**MLR**	**5.80**	**(1.45-23.79)**	**0.0133**	**―**	**―**	**―**

Abbreviations: OR, odds ratio; CI, confidence interval; PSA, prostate-specific antigen; PSAD, PSA density; WBC, white blood cell; CRP, C-reactive protein; NLR, neutrophil-to-lymphocyte ratio; MLR, monocyte-to-lymphocyte ratio.

When the regression coefficients of the optimum model were estimated, the following model for predicting high Gleason score cancer by biopsy was calculated: *P* = [1+exp (7.738-0.050×age-6.754×PSAD-0.263×monocyte fraction)]^-1^. The AUC by age, PSAD, and serum monocyte fraction was 0.778 (95% CI 0.732-0.818), whereas that by PSA was 0.586 (95% CI 0.535-0.634) in men with PSA < 10 ng/ml (Figure 1b). The sensitivity and specificity of the model at the best cutoff were 62.3% and 81.7%, respectively.

Serum monocyte fraction was also significantly higher in men with Gleason score≥8 prostate cancer than in men with negative biopsy and Gleason score≤7 prostate cancer in the cohort of men with PSA < 10 ng/ml (Supplementary Table 3). Similarly, univariate and multivariate analysis showed that serum monocyte fraction was significantly associated with Gleason score≥8 prostate cancer (Supplementary Table 4).

## DISCUSSION

Although PSA is undoubtedly a standard serum biomarker for the detection of prostate cancer, it is problematic that the diagnosis of prostate cancer by serum PSA alone often results in overdiagnosis or overtreatment. [11] Recently, active surveillance has become one of treatment options for clinically insignificant prostate cancer in many countries. [12–14] Although there are several criteria of active surveillance for insignificant prostate cancer, it was reported that such criteria seem to have a lower ability to predict insignificant cancer in Asian men. [15] The discovery of convenient biomarkers for the detection of clinically significant cancer before needle biopsy is desired.

In the present study, the monocyte fraction of WBCs was associated with high Gleason score prostate cancer and was one of the significant predictors as were age and PSAD. In the men with PSA < 10 ng/ml, monocyte fraction was similarly associated with, and one of the independent predictors for, high Gleason score prostate cancer. MLR was not a significant predictor of high Gleason score cancer in a stepwise multiple logistic regression analysis.

This is the first study, to our knowledge, to show an association of serum monocyte fraction of WBCs with high Gleason score prostate cancer. The serum monocyte fraction was significantly increased in the patients with high Gleason score cancer even in the group of men with a PSA < 10 ng/ml who were predicted to have low tumor volume from the PSA value alone. The cause of the increased monocyte fraction in patients with high Gleason score prostate cancer is not clearly defined. *In vitro* studies of prostate cancer reported that monocyte-induced cancer cell invasion mediates chemokine ligand 2 (monocyte chemotactic protein-1) and nuclear factor-κB activity [16] and that tumor stroma-derived factors skew monocyte to dendritic cell differentiation toward a suppressive phenotype [17]. Tumor-associated macrophages (TAMs), which are supposed to be derived from serum monocytes, may interact with tumor cells to promote cancer progression by producing various cytokines and chemokines. [18] We reported that the infiltration of TAMs in prostate biopsy specimens was predictive of disease progression of prostate cancer after hormonal therapy. [19] The creation of TAMs in the tumor microenvironment may be accelerated by high numbers of serum monocytes. Inversely, the large numbers of various cytokines and chemokines produced by cancer cells may have effects on the number of serum monocytes.

There are several limitations in this study. First, the number of patients diagnosed as having low Gleason score prostate cancer (i.e., Gleason score of ≤6) is low. Second, although this study included men in two hospitals, we did not undergo a retrospective central pathology review of the specimens. Finally, we decided to use the Gleason score determined from a needle biopsy specimen, which might differ from that determined from a prostatectomy specimen. [20]

We expect that the serum monocyte fraction of WBC may become one of the candidate biomarkers for clinically significant prostate cancer in combination with age, PSAD, and other biomarkers. Further investigations and validations are needed to ascertain whether the serum monocyte fraction is one of the useful biomarkers of prostate cancer and to elucidate the mechanism of the association of serum monocytes with the progression of prostate cancer. It should be also explored if serum monocyte fraction is associated with the prognosis of prostate cancer patients.

## CONCLUSIONS

The serum monocyte fraction of WBCs was significantly increased in patients with high Gleason score (Gleason score ≥7) prostate cancer. Serum monocytes were suggested to be associated with the progression of prostate cancer, and further investigations will be necessary to elucidate the mechanism of this association.

## MATERIALS AND METHODS

### Patients and subjects

Transrectal ultrasound-guided 12-core prostate needle biopsy without MRI fusion was performed in 966 men with suspicion of prostate cancer (643 men at Osaka University Hospital from January, 2010 to December, 2014 and 323 men at Osaka General Medical Center from January, 2010 to September, 2011). Indication of prostate needle biopsy was abnormal serum PSA level or abnormal radiological findings on MRI or PET-CT. In several cases, additional several target biopsies were performed in men with abnormal MRI images. Twenty-three repeat biopsies were included. Blood tests were performed before prostate needle biopsy. We retrospectively assessed age, serum PSA, prostate volume, WBC count, each fraction of WBCs (neutrophils, lymphocytes, monocytes, basophils, and eosinophils) and CRP level before biopsy for associations with biopsy findings (positive or negative and Gleason Score). PSAD was calculated as serum PSA level divided by prostate volume. NLR and MLR were calculated as each neutrophil fraction and monocyte fraction divided by lymphocyte fraction. We decided Gleason grades by the reports from the pathologists in each hospital based on classifications at the 2005 ISUP Consensus Conference. This study was approved by the institutional review board of Osaka University Hospital and Osaka General Medical Center.

### Statistical analysis

Results are presented as the median (range). High Gleason score and low Gleason score prostate cancer was respectively defined as a Gleason score ≥7 and ≤6 in biopsy findings. Patients’ characteristics were analyzed using the Kruskal-Wallis test in three groups, and Mann-Whitney *U* test in two groups. Univariate analysis for predicting positive biopsy or high Gleason score prostate cancer was identified by logistic regression analysis in which variables of age, PSA, PSAD, WBC count, fractions of each of the WBC types, NLR, MLR, and CRP level were used. Stepwise multiple logistic regression analysis was performed with the variables that were significantly associated with positive biopsy or high Gleason score cancer in the univariate analysis. The variables that were significantly associated with high Gleason score cancer in the multivariate analysis were then entered into an optimum model for predicting high Gleason score cancer. All analyses were performed by JMP Pro 11.2.0 (SAS Institute). Statistical significance was determined as *p* < 0.05.

## SUPPLEMENTARY MATERIALS FIGURES AND TABLES



## References

[1] Polascik TJ, Oesterling JE, Partin AW (1999). Prostate specific antigen: a decade of discovery-what we have learned and where we are going. J Urol.

[2] Saini S (2016). PSA and beyond: alternative prostate cancer biomarkers. Cell Oncol (Dordr).

[3] Nakajima K, Heilbrun LK, Smith D, Hogan V, Raz A, Heath E (2017). The influence of PSA autoantibodies in prostate cancer patients: a prospective clinical study-II. Oncotarget.

[4] Fujita K, Hayashi T, Matsuzaki K, Nakata W, Kawashima A, Ujike T, Nagahara A, Tsuchiya M, Kobayashi Y, Uemura M, Miyoshi E, Nonomura N (2016). Decreased fucosylated PSA as a urinary marker for high Gleason score prostate cancer. Oncotarget.

[5] Fujita K, Imamura R, Tanigawa G, Nakagawa M, Hayashi T, Kishimoto N, Hosomi M, Yamaguchi S (2012). Low serum neutrophil count predicts a positive biopsy. Prostate Cancer Prostatic Dis.

[6] Fujita K, Hosomi M, Nakagawa M, Tanigawa G, Imamura R, Uemura M, Nakai Y, Takayama H, Yamaguchi S, Nonomura N (2014). White blood cell count is positively associated with benign prostatic hyperplasia. Int J Urol.

[7] Liu ZQ, Chu L, Fang JM, Zhang X, Zhao HX, Chen YJ, Xu Q (2014). Prognostic role of C-reactive protein in prostate cancer: a systematic review and meta-analysis. Asian J Androl.

[8] Rocha P, Morgan CJ, Templeton AJ, Pond GR, Naik G, Sonpavde G (2014). Prognostic impact of C-reactive protein in metastatic prostate cancer: a systematic review and meta-analysis. Oncol Res Treat.

[9] Kawahara T, Fukui S, Sakamaki K, Ito Y, Ito H, Kobayashi N, Izumi K, Yokomizo Y, Miyoshi Y, Makiyama K, Nakaigawa N, Yamanaka T, Yao M (2015). Neutrophil-to-lymphocyte ratio predicts prostatic carcinoma in men undergoing needle biopsy. Oncotarget.

[10] Wang L, Long W, Li PF, Lin YB, Liang Y (2015). An elevated peripheral blood monocyte-to-lymphocyte ratio predicts poor prognosis in patients with primary pulmonary lymphoepithelioma-like carcinoma. PLoS One.

[11] Lippi G, Montagnana M, Guidi GC, Plebani M (2009). Prostate-specific antigen-based screening for prostate cancer in the third millennium: useful or hype?. Ann Med.

[12] Chen RC, Rumble RB, Loblaw DA, Finelli A, Cooperberg MR, Morgan SC, Tyldesley S, Haluschak JJ, Tan W, Justman S, Jain S (2016). Active surveillance for the management of localized prostate cancer (Cancer Care Ontario Guideline): American Society of Clinical Oncology Clinical Practice Guideline endorsement. J Clin Oncol.

[13] Amin MB, Lin DW, Gore JL, Srigley JR, Samaratunga H, Egevad L, Rubin M, Nacey J, Carter HB, Klotz L, Sandler H, Zietman AL, Holden S (2014). The critical role of the pathologist in determining eligibility for active surveillance as a management option in patients with prostate cancer: consensus statement with recommendations supported by the College of American Pathologists, International Society of Urological Pathology, Association of Directors of Anatomic and Surgical Pathology, the New Zealand Society of Pathologists, and the Prostate Cancer Foundation. Arch Pathol Lab Med.

[14] Van del Kwast T, Bubendorf L, Mazerolles C, Raspollini MR, Van Leenders GJ, Pihl CG, Kujala P (2013). Guidelines on processing and reporting of prostate biopsies: the 2013 update of the pathology committee of the European Randomized Study of Screening for Prostate Cancer (ERSPC). Virchows Archiv.

[15] Yamada Y, Sakamoto S, Sazuka T, Goto Y, Kawamura K, Imamoto T, Nihei N, Suzuki H, Akakura K, Ichikawa T (2016). Validation of active surveillance criteria for pathologically insignificant prostate cancer in Asian men. Int J Urol.

[16] Lindholm PF, Sivapurapu N, Jovanovic B, Kajdacsy-Balla A (2015). Monocyte-induced prostate cancer cell invasion is mediated by chemokine ligand 2 and nuclear factor-κB activity. J Clin Cell Immunol.

[17] Spary LK, Salimu J, Webber JP, Clayton A, Mason MD, Tabi Z (2014). Tumor stroma-derived factors skew monocyte to dendritic cell differentiation toward a suppressive CD14+PD-L1+ phenotype in prostate cancer. Oncoimmunology.

[18] Mantovani A, Bottazzi B, Colotta F, Sozzani S, Ruco L (1992). The origin and function of tumor-associated macrophages. Immunol Today.

[19] Nonomura N, Takayama H, Nakayama M, Nakai Y, Kawashima A, Mukai M, Nagahara A, Aozasa K, Tsujimura A (2011). Infiltration of tumour-associated macrophages in prostate biopsy specimens is predictive of disease progression after hormonal therapy for prostate cancer. BJU Int.

[20] Hoogland AM, Böttcher R, Verhoef E, Jenster G, van Leenders GJ (2016). Gene-expression analysis of gleason grade 3 tumor glands embedded in low- and high-risk prostate cancer. Oncotarget.

