# Supplementary material for: Serum monocyte fraction of white blood cells is increased in patients with high Gleason score prostate cancer
Source: Oncotarget. 2016 Nov 3;8(21):35255–61. doi: 10.18632/oncotarget.13052 (PMC5471051; doi:10.18632/oncotarget.13052)
Supplement: Supplementary file 1 [file oncotarget-08-35255-s001.pdf]

Serum monocyte fraction of white blood cells is increased in patients with high Gleason score prostate cancer

Supplementary Material

Supplementary Table 1: Characteristics and biopsy findings of all men (comparison of the men with positive biopsy to those with negative biopsy)

Characteristic	NC		LG+HG		p-Value
Number	413		131+422		
**Age (years)**	**67**	**(44-84)**	**71**	**(36-89)**	**<0.0001**
**PSA (ng/ml)**	**6.39**	**(0.36-935)**	**10.99**	**(1.49-10138)**	**<0.0001**
**Prostate volume (cm^3)**	**40.0**	**(10.0-150.0)**	**28.1**	**(7.3-160.0)**	**<0.0001**
**PSAD ($ng/ml/cm^3$)**	**0.17**	**(0.01-12.99)**	**0.40**	**(0.03-330.87)**	**<0.0001**
WBC count (/µl)	5850	(2390-13870)	5700	(3170-12400)	0.1613
Neutrophil fraction (%)	61.2	(34.2-91.5)	60.1	(27.0-87.3)	0.3666
Lymphocyte fraction (%)	28.8	(4.2-55.8)	28.1	(9.2-65.4)	0.6325
**Monocyte fraction (%)**	**6.7**	**(2.5-13.2)**	**7.3**	**(1.8-17.1)**	**<0.0001**
Basophil fraction (%)	0.6	(0-3.1)	0.5	(0-4.1)	0.5051
Eosinophil fraction (%)	2.5	(0-18.1)	2.3	(0-15.5)	0.5024
CRP (mg/dl)	0.05	(0-8.55)	0.05	(0-16.75)	0.1293
NLR	2.12	(0.61-21.79)	2.12	(0.41-8.39)	0.9448

**MLR**	**0.23**	**(0.08-1.49)**	**0.26**	**(0.04-1.25)**	**<0.0001**

NC, no cancer; LG, low Gleason score cancer; HG: high Gleason score cancer; PSA, prostate-specific antigen; PSAD, PSA density; WBC, white blood cell; CRP, C-reactive protein; NLR, neutrophil-to-lymphocyte ratio; MLR, monocyte-to-lymphocyte ratio.

Supplementary Table 2: Univariate and multivariate analyses for predicting positive biopsy in all men (n=966)

	Univariate analysis			Multivariate analysis		
Variable	OR	95% CI	p-Value	OR	95% CI	p-Value
**Age (years)**	**1.07**	**(1.05-1.09)**	**<0.0001**	**1.06**	**(1.03-1.08)**	**<0.0001**
**PSA (ng/ml)**	**1.02**	**(1.01-1.03)**	**<0.0001**	**0.95**	**(0.94-0.96)**	**<0.0001**
**PSAD (ng/ml/cm^3)**	**17.80**	**(9.22-36.45)**	**<0.0001**	**186.29**	**(64.23-590.32)**	**<0.0001**
WBC count (/µl)	1.00	(1.00-1.00)	0.161			
Neutrophil (%)	0.99	(0.98-1.01)	0.291			

Lymphocyte (%)	1.00	(0.98-1.01)	0.793			
**Monocyte (%)**	**1.24**	**(1.16-1.33)**	**<0.0001**	**1.17**	**(1.08-1.27)**	**0.0001**
Basophil (%)	0.93	(0.65-1.32)	0.666			
Eosinophil (%)	0.98	(0.93-1.04)	0.529			
**CRP (mg/dl)**	**1.23**	**(1.07-1.49)**	**0.0024**	—	—	—
NLR	0.97	(0.88-1.06)	0.510			
**MLR**	**6.02**	**(2.19-17.59)**	**0.0004**	—	—	—

OR, odds ratio; CI, confidence interval; PSA, prostate-specific antigen; PSAD, PSA density; WBC, white blood cell; CRP, C-reactive protein; NLR, neutrophil-to-lymphocyte ratio; MLR, monocyte-to-lymphocyte ratio.

Supplementary Table 3: Characteristics and biopsy findings of men with PSA <10 ng/ml (comparison of the men with negative biopsy and those with Gleason score ≤7 to those with Gleason score ≥8)

Characteristic	NC+GS≤7		GS≥8		p-Value
Number	322+186		63		
**Age (years)**	**67**	**(36-82)**	**71**	**(53-87)**	**0.0002**
**PSA (ng/ml)**	**5.80**	**(0.36-9.99)**	**6.84**	**(1.49-9.80)**	**0.0008**
**Prostate volume (cm^3)**	**31.9**	**(7.3-150.0)**	**24.0**	**(7.8-77.0)**	**<0.0001**
**PSAD (ng/ml/cm^3)**	**0.17**	**(0.01-0.82)**	**0.28**	**(0.04-0.70)**	**<0.0001**
**WBC count (/μl)**	**5640**	**(2390-13870)**	**5150**	**(3170-10280)**	**0.0316**
**Neutrophil fraction (%)**	**60.9**	**(27.0-91.5)**	**57.1**	**(39.4-78.0)**	**0.0300**
Lymphocyte fraction (%)	28.5	(4.2-65.4)	31.3	(9.2-48.7)	0.3027
**Monocyte fraction (%)**	**6.7**	**(1.8-13.2)**	**8.2**	**(4.9-15.4)**	**<0.0001**
Basophil fraction (%)	0.6	(0-4.1)	0.6	(0.1-1.9)	0.9001
Eosinophil fraction (%)	2.5	(0-15.7)	2.4	(0-14.2)	0.4336
CRP (mg/dl)	0.05	(0-6.51)	0.04	(0-6.41)	0.7140
NLR	2.12	(0.41-21.79)	1.78	(0.89-8.23)	0.1264
**MLR**	**0.23**	**(0.08-0.97)**	**0.29**	**(0.13-1.25)**	**0.0032**

NC, no cancer; GS, Gleason score; PSA, prostate-specific antigen; PSAD, PSA density; WBC, white blood cell; CRP, C-reactive protein; NLR, neutrophil-to-lymphocyte ratio; MLR, monocyte-to-lymphocyte ratio.

Supplementary Table 4: Univariate and multivariate analyses for predicting Gleason score ≥8 in men with PSA <10 ng/ml (n=571)

	Univariate analysis			Multivariate analysis		
Variable	OR	95% CI	p-Value	OR	95% CI	p-Value
**Age (years)**	**1.08**	**(1.04-1.13)**	**<0.0001**	**1.07**	**(1.03-1.12)**	**0.0016**
**PSA (ng/ml)**	**1.26**	**(1.10-1.46)**	**0.0007**	—	—	—
**PSAD (ng/ml/cm^3)**	**285.82**	**(46.01-1947.56)**	**<0.0001**	**340.63**	**(48.90-2557.11)**	**<0.0001**
**WBC count** (/μl)	**1.00**	**(1.00-1.00)**	**0.0483**	—	—	—
**Neutrophil (%)**	**0.97**	**(0.94-1.00)**	**0.0355**	—	—	—
Lymphocyte (%)	1.01	(0.98-1.04)	0.512			
**Monocyte (%)**	**1.47**	**(1.30-1.69)**	**<0.0001**	**1.45**	**(1.26-1.67)**	**<0.0001**
Basophil (%)	0.97	(0.45-1.86)	0.942			

Eosinophil (%)	1.04	(0.92-1.15)	0.528			
CRP (mg/dl)	1.22	(0.84-1.64)	0.260			
NLR	0.96	(0.75-1.14)	0.668			
**MLR**	**20.23**	**(3.76-110.59)**	**0.0006**	—	—	—

OR, odds ratio; CI, confidence interval; PSA, prostate-specific antigen; PSAD, PSA density; WBC, white blood cell; CRP, C-reactive protein; NLR, neutrophil-to-lymphocyte ratio; MLR, monocyte-to-lymphocyte ratio.
